# Effect on Pain Following One Session of Botulinum Toxin Type A in Patients With Jaw Myalgia: A Randomised Double‐Blind Controlled Multicentre Pilot Study

**DOI:** 10.1111/joor.13915

**Published:** 2024-12-29

**Authors:** Ava Minston, Helene Abrahamsson, Peter Abrahamsson, Erik Lindfors, Eva Nohlert, Daniel Ovesson, Negin Yekkalam, Göran Isacsson

**Affiliations:** ^1^ Department of Orofacial Pain and Jaw Function, Postgraduate Dental Education Center Public Dental Health Service Örebro Sweden; ^2^ Department of Orofacial Pain and Jaw Function Aqua Dental Specialist Clinic Stockholm Sweden; ^3^ The Maxillofacial Unit Halmstad Hospital Halmstad Sweden; ^4^ Department of Oral and Maxillofacial Surgery and Oral Medicine Malmö University Malmö Sweden; ^5^ Department of Orofacial Pain and Jaw Function Public Dental Health Service Uppsala Sweden; ^6^ Region Vastmanland—Uppsala University, Centre for Clinical Research Västmanland Hospital Västerås Sweden; ^7^ Department of Orofacial Pain and Jaw Function Karlstad Hospital Karlstad Sweden; ^8^ Department of Orofacial Pain and Jaw Function Västmanland County Hospital Västerås Sweden; ^9^ Department of Orofacial Pain and Jaw Function Umeå University Umeå Sweden; ^10^ Faculty of Medicine and Health University Health Care Research Center Örebro Sweden

**Keywords:** botulinum toxin type A, myofascial pain, orofacial pain, temporomandibular disorders, treatment

## Abstract

**Background:**

Botulinum toxin Type A (BTX‐A) is increasingly used in the management of myofascial pain; however, the evidence to support this treatment modality is still inconclusive.

**Objective:**

To evaluate the change in days with functional jaw pain after one session of BTX‐A or placebo injections into the masseter and temporalis muscles in subjects with jaw myalgia. The hypothesis was that BTX‐A is effective in reducing the number of days with functional jaw pain.

**Methods:**

This was a randomised, double‐blind, parallel group study in subjects with jaw myalgia. After randomisation, BTX‐A or placebo injections were made in the masseter and temporalis muscles. The number of days with jaw pain on function was evaluated after 2 months. Adverse events were registered.

**Results:**

Twenty‐three subjects were randomly assigned to BTX‐A and 22 to placebo. Between baseline and the 2‐month follow‐up, the number of days with jaw pain at function changed from a median (interquartile range) of 14 (4) to 10.5 (6) days in the BTX‐A group and from 14.0 (1.5) to 14 (5) in the saline group (*p* < 0.585). Adverse events were mild and transient and similar in the two groups.

**Conclusion:**

A single treatment of BTX‐A injections in the masseter and temporalis muscles was not effective in treating jaw myalgia. However, an adequately powered study might provide sufficient support for efficacy.

## Graphical abstract

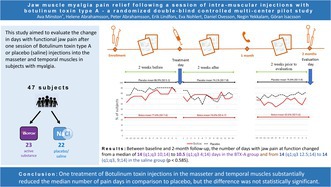

## 1 Introduction

Temporomandibular disorders (TMD) are the second most common musculoskeletal condition, after chronic low back pain [1] and refer to pain and dysfunction in the temporomandibular system and its muscles, joints and associated structures. Diagnostic Criteria for Temporomandibular Disorders (DC/TMD) were introduced in 2014 and are now used worldwide to classify TMD [2].

The prevalence of TMD symptoms is 4%–15% among adults where myalgia is the most common form of TMD [1, 3, 4] affecting about half of the subjects presenting in TMD clinics [5], and is defined by pain in a masticatory structure that is modified by jaw movement, function or parafunction and familiar pain in masticatory muscle(s) at either muscle palpation or at maximum opening.

Myalgia is usually treated with conservative methods including occlusal, physical and pharmacologic therapies. However, there is no evidence regarding which conservative treatment is the most beneficial mainly because of a lack of randomised controlled trials.

Botulinum toxin Type A (BTX‐A) is a neurotoxin isolated from the bacterium *Clostridium botulinum* . Injected into a muscle, BTX‐A's action occurs in the presynaptic junction by blocking the release of acetylcholine, resulting in weakening the muscle. The clinical effect occurs 3–7 days after administration and diminishes after 3 months, as sprouting causes the formation of new synaptic connections [6]. It has also been reported that BTX‐A blocks the release of inflammatory mediators including glutamate and substance P, thus having an antinociceptive effect [7].

The use of BTX‐A has expanded significantly in recent years, and it has gained considerable attention as a treatment for pain, but often off‐label. There have been interesting results in treating other muscle conditions such as cervical dystonia [8] and chronic migraine [9].

Despite its promising mechanism of action, BTX‐A's efficacy in treating myalgia remains uncertain, with inconclusive evidence from systematic reviews and meta‐analyses due to study heterogeneity and methodological limitations [10, 11, 12]. However, other meta‐analyses and systematic reviews have shown a significant reduction in jaw muscle pain following one session of BTX‐A treatment [13, 14].

The National Board of Health and Welfare in Sweden does not recommend the use of BTX‐A for treating myofascial pain because of inconclusive scientific evidence [15]. The key publications describing BTX‐A for the treatment of myofascial pain use reduction in pain intensity as the primary measure of efficacy. However, this approach may be a blunt instrument in the evaluation of amelioration of chronic pain.

Therefore, there is a need to explore BTX‐A treatment effects of myofascial pain in relation to side effects using a set of measures that will allow hypothesis generation.

The aim of this study was to evaluate the change of days with functional pain after one session of BTX‐A or placebo injections in the masseter and temporalis muscle, as well as performing exploratory analysis on a series of variables to identify those that display adequate sensitivity for use in later dimensioned randomised controlled trials.

## 2 Materials and Methods

### 2.1 Study Design

The study was designed as a double‐blind randomised controlled hypothesis generation two‐arm parallel group trial in subjects referred to a specialist dental clinic for treatment of jaw muscle myalgia. The study team at each centre consisted of a dentist and a study nurse. Before the start of the study, all investigators were instructed and calibrated both in the injection technique and in the clinical examination.

Subjects visited the clinic at three predefined occasions, and there was one scheduled telephone call. Visit 1: Enrolment, subject information and informed consent. After checking, the inclusion and exclusion criteria the subject was assigned an enrolment number. Clinical examination was performed by the dentist to check inclusion and exclusion criteria. Questionnaires and diaries were handed out and the subject was instructed. Visit 2: Two to 3 weeks (after enrolment visit). A baseline clinical examination was performed. The questionnaire and diary were returned. After checking the inclusion and exclusion criteria, the intervention substance was prepared according to the randomisation code and the dentist performed the treatment. The subject was instructed to complete a new set of questionnaires to be used 2 weeks after the intervention. Adverse events were registered. The subject returned the questionnaires and diaries by surface mail after 2 weeks. Telephone evaluation: 1 month (window 1 week) after Visit 2. Telephone call. Adverse events were registered. Visit 3: 2 months (window 2 weeks) after Visit 2. The dentist collected the questionnaires and diaries, and a clinical examination was performed. Adverse events were registered. The subject was then completed within the scope of the study. Any further treatment was completed outside the study protocol.

Baseline data (point estimates) were collected directly before intervention and at the 2‐month follow‐up visit.

### 2.2 Subject Selection

This study was primarily targeted to include a total of 48 subjects from four orofacial pain specialist clinics in Sweden. Due to the COVID‐19 pandemic the recruitment of subjects was delayed, and two additional investigational sites were opened and calibrated in the same manner as the original centres.

Subjects referred to one of the specialist clinics were screened for inclusion. Those who met the criteria for a myalgia diagnosis according to DC/TMD [2] were the target group for this study. Inclusion criteria were age ≥ 18 years, ≥ 3‐months of complaints of jaw and/or face pain, a diagnosis of myalgia according to DC/TMD, a demonstrated proficiency in verbal and written Swedish, and able to provide written consent to participate in the study. Exclusion criteria were polyarthritis/connective tissue disease, fibromyalgia or other generalised pain, neurological disorders, whiplash‐associated disorders, ongoing virus or bacterial infection, ongoing dental treatment, botulinum toxin injection in the jaws or face in the past 6 months, complex psychiatric or psychological profile, institutional resident, employee at the trial clinic, hypersensitivity to botulinum toxin or human albumin, serious health conditions according to the examiner's assessment, presence of infection at the proposed injection site(s), pregnancy or lactating, fertile women not on contraception.

In diagnosing myalgia, the history must be positive for both of the following: Pain in the jaw, temple, in front of the ear or in the ear in the past 30 days and pain modified by jaw movement, function or parafunction. In addition, examination of the temporalis or masseter muscles must confirm one of the following: Confirmation of pain location in the area of the temporalis or masseter muscle and/or familiar muscle pain at palpation or maximum opening.

### 2.3 Randomisation, Blinding and Monitoring

A randomisation list was generated by computer (http://randomization.com/) and balanced with as many subjects in the treatment group as in the control group. Randomisation, which was sealed in individual envelopes was performed at the Centre for Clinical Research, Västerås by a person not attached to the project. The randomisation list was kept locked up until ‘clean file’ was declared. A study monitor checked all records and collected all case report forms before data analysis.

### 2.4 Study Treatment

The investigational product was Botox 100 Allergan units ( *Clostridium botulinum* neurotoxin Type A hemagglutinin complex, AbbVie AB, Solna, Sweden). One treatment session consisted of a total dose of 100 Allergan units distributed at 14 intramuscular injection sites. Dilutant was 0.9% sodium chloride for parenteral injection.

The reference substance (placebo) was 0.9% sodium chloride for parenteral injection. The BTX‐A‐loaded syringes were prepared by dissolving 100 units of freeze‐dried Botox in 2.0 mL of room‐temperature sterile isotonic saline thus providing syringes with a concentration of 5 units per 0.1 mL. The preparation of the solution was made directly before use. The reference substance was prepared in identical syringes and volumes. After the investigator checked alignment to the inclusion/exclusion criteria, the study nurse prepared the syringes in a separate room according to the randomisation envelope information. The syringes were then provided to the investigator, ensuring that both the investigator and study subject were blinded to the syringe contents.

**FIGURE 1:**
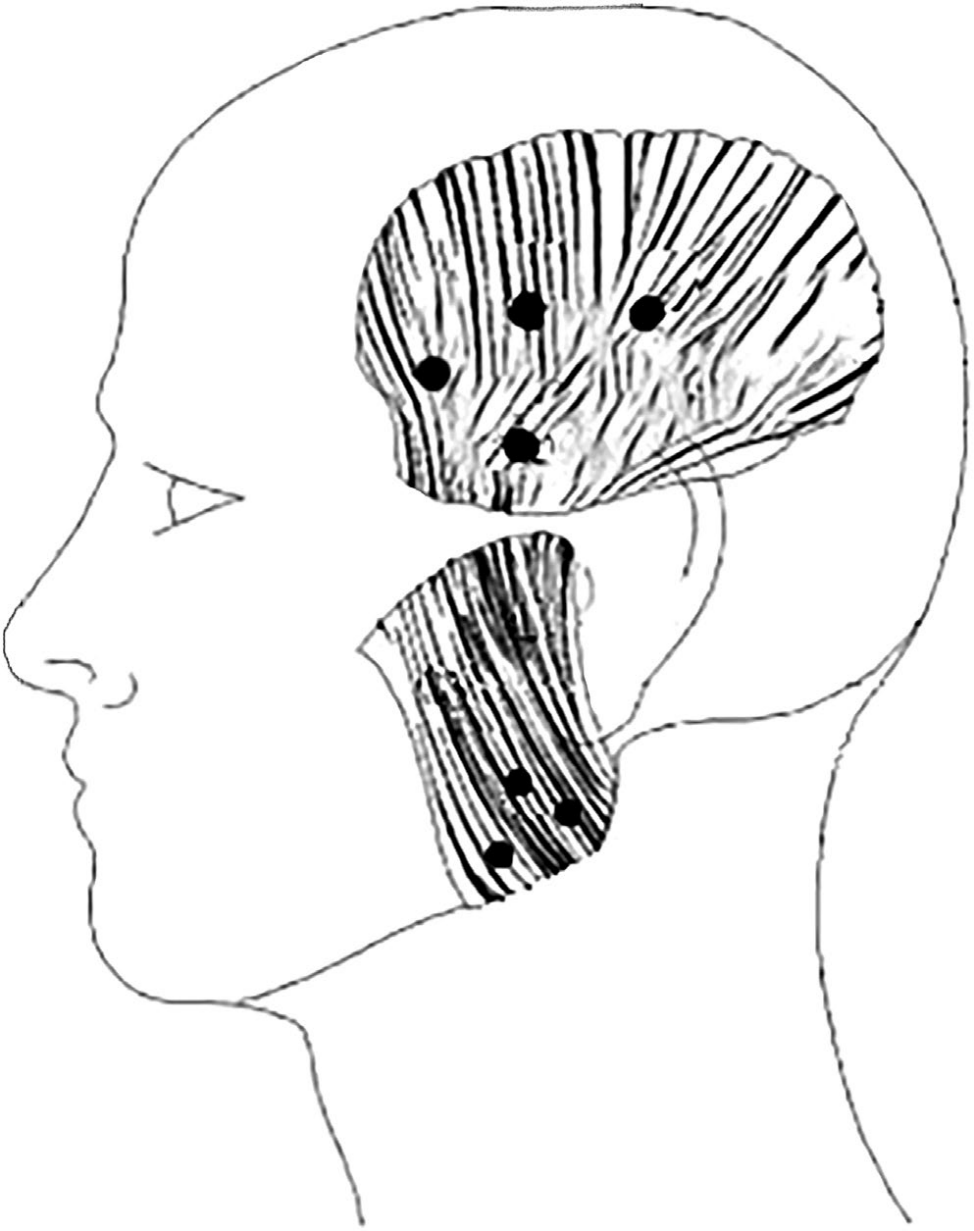
Injection sites modified from BTX‐A packet insert [16].

Injection treatment was made at both the bilateral and unilateral sites of myalgia. Clenching the teeth, thus activating the jaw muscles helped to localise the injection sites in the temporalis and the masseter muscles. Injections were made at four sites in each temporalis muscle according to the manufacturer's recommendation for chronic migraine and at three sites in each masseter muscle (Figure 1).

The test substance was injected using a gauge 0.3 mm needle. A total dose of 40 units was injected at the eight sites of the temporalis muscles (5 units at each site), and a total dose of 60 units was injected in the six sites of the masseter muscles (10 units at each site), that is, a total dose of 100 units.

### 2.5 Concomitant Treatment

Other medication necessary for the subject's care could be given at the investigator's discretion. All subjects were allowed to take analgesics if necessary. The type of analgesics was recorded daily in the diary. Those who used oral appliances at the time of enrolment continued with their appliances at the same routine throughout the study time. Any new treatment was not allowed during the study period. If a subject still had complaints from the jaw at the evaluation visit, additional treatment was offered according to the clinic's routine, but outside the study protocol and after all study data was recorded.

### 2.6 Outcomes

The primary variable was the change in frequency (number) of days with functional jaw pain. The base for evaluation was the 2 weeks prior to intervention and the 2 weeks prior to evaluation visit. The data were obtained from a diary.

The Graded Chronic Pain Scale (GCPS) was used for recording pain intensity where Characteristic Pain Intensity (CPI) was the mean value of the current, average as well as the worst pain intensity the previous month. Change of pain intensity was recorded at maximal opening and at jaw rest using the numeric rating scale 0–10 (NRS) with the end definitions ‘no pain’ and ‘worst pain imaginable’. Jaw functional problems were traced using the 8‐item Jaw Functional Limitation Scale (JFLS‐8), with possible mean scores ranging from 0 (no limitation) to 10 (severe limitation) [17]. Patient Health Questionnaire‐9 (PHQ‐9) is a multipurpose instrument used for screening, diagnosing and measuring the severity of depression, with possible scores ranging from 0 to 27.

At the follow‐up visit, subjects rated the change in their overall status since beginning the study treatment using the Patient Global Impression of Change (PGIC) scale, a 7‐point instrument ranging from ‘very much improved’ to ‘very much worse’ [18]. The change of maximum mouth opening (distance between the incisal edges plus vertical overlap at the left central incisor) was measured as maximum pain free opening, maximum unassisted opening and maximum assisted opening. The change in palpation tenderness (yes/no) over m. masseter and m. temporalis bilaterally was captured according to the DC/TMD protocol.

### 2.7 Adverse Events

Patients were asked to list any health‐related event and its duration in the diaries, at the follow‐up phone call, as well as at clinical visits. The investigators also registered observed health events in the clinic. Before leaving the clinic after intervention and at the follow‐up visit as well as at the phone call treatment visit the subject was asked if they experienced any health incidents. Each adverse event (AE) was classified by the investigator as mild, moderate or severe, and the investigator was also responsible for determining whether there was a causal relationship between the AE and use of the investigational product. All AEs were categorised either as likely related, possibly related or not related.

### 2.8 Statistics

Tolerability and safety evaluation was based on all subjects exposed to the investigational product. The exception was one subject with a severe protocol violation by means of receiving several other treatments directly after injection of the investigational product. Any adverse event concerning that subject is presented separately. The data from that subject were not included in the efficacy summary analysis.

The primary effect analysis was based on all subjects who received any dose of the study drug, that is, the intention to treat (ITT) population (with the above exception). Categorical variables are presented as number and percentage. Continuous variables are presented as mean and standard deviation (SD) if normally distributed and median and interquartile range if not normally distributed. Nonparametric statistical significance tests are Pearson chi‐square test/Fisher's exact test, Wilcoxon Mann–Whitney *U*‐test and Wilcoxon signed rank test. Parametric statistical significance tests are Student's *t*‐test and paired *t*‐test. Statistically significant differences accepted at *p* < 0.05 (two‐sided). Exploratory descriptive analyses and sub analyses were also performed.

There is no solid basis of published data for a power calculation of the primary measure. For that reason, no power calculation was made, but a reasonable large group of subjects (*n* = 48) was planned to be included forming the basis for hypothesis generation and later being a base for future randomised controlled trials with sufficient power.

### 2.9 Ethics

The study was performed in accordance with the study protocol, ICH‐GCP E6 (R2), the latest version of the Declaration of Helsinki and applicable regulatory requirements. The subjects received complete and adequate oral and written information about the study and its performance, purpose, risks and benefits. All subjects gave written consent before enrolment. The Swedish Ethical Review Authority (Etikprövningsmyndigheten) approved the study 18th May 2020, EPM #2020‐00940.

## 3 Results

**FIGURE 2:**
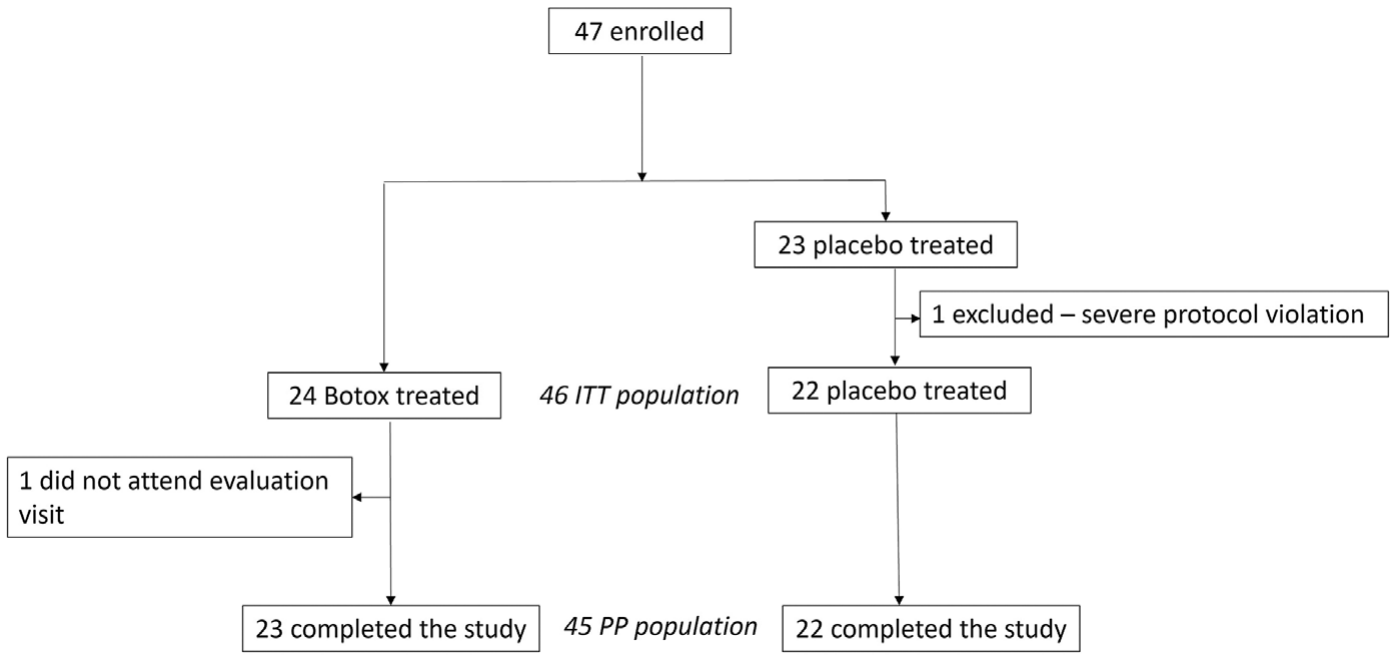
Flow chart of study subjects.

At enrolment, informed consent was obtained for a total of 47 subjects, none of whom withdrew their consent before randomisation. One subject in the placebo group underwent several other treatments temporally close to the study treatment, resulting in a severe protocol deviation and was consequently not included in any analysis. The ITT population comprised 24 subjects treated with BTX‐A and 22 treated with placebo. One of the BTX‐A treated subjects did not present for evaluation at the end of the study period and their data was handled as ‘baseline observation carried forward’. Figure 2 illustrates the summary profile of the trial including both ITT and Per‐Protocol (PP) populations. Baseline demographics and measures showed minor nonsignificant differences between the groups (Table 1).

**TABLE 1:** Demographic characteristics of BTX‐A‐ and placebo‐treated subjects at baseline.

	BTX‐A (*n* = 24)	Placebo (*n* = 23)	*p*
Age, years^a^	37.3 (10.7)	33.8 (10.8)	0.274
BMI^a^	26.2 (4.4)	24.5 (5.2)	0.247
Female gender^b^	20 (83)	20 (91)	0.667
Number of days with pain at function^c,^ ^†^	14 (4)	14 (1.5)	0.227
Number of days with pain at rest^c^	14 (1.7)	14 (3.2)	0.805
Number of days with jaw pain at function or rest^c^	14 (1)	14 (0)	0.089
JFLS‐8^c^	8.5 (12.5)	12.0 (17.7)	0.327
GCPS	2 (1–2)	1 (1–2)	0.262
GCPS grade^b^			
I	10 (42)	13 (59)	0.682
II	10 (42)	7 (32)	
III	2 (8)	1 (5)	
IV	2 (8)	1 (5)	
PHQ‐9^c^	5.5 (6.7)	5.0 (7.5)	0.882
Pain at maximum opening^a,^ ^‡^	4.8 (2.3)	4.7 (2.4)	0.828
Pain at jaw rest^a,^ ^‡^	3.9 (1.6)	3.4 (1.8)	0.272

*Note:* Data are ^a^mean (SD), ^b^number of subjects (%) or ^c^median (interquartile range). *p* values tested by ^a^Student's *t*‐test, ^b^Pearson chi‐square test/Fisher's exact test or ^c^Mann–Whitney *U*‐test.

Abbreviations: GCPS, Graded Chronic Pain Severity Scale; JFLS‐8, Jaw Functional Limitation Scale‐8 items; PHQ‐9, Subject Health Questionnaire‐9.

^†^ Pain in the jaw when eating, chewing, yawing, etc.

^‡^ Numeric rating scale (0–10) with the end definitions ‘No pain’ and ‘Worst pain imaginable’.

The duration between baseline and the end‐of‐study visit was a mean 86 days (SD 18) and 98 days (SD 41) for the BTX‐A‐ and placebo‐treated, respectively. Concomitant use of an occlusal appliance was seen in 14 subjects in the BTX‐A group and 13 in the placebo group. In both the BTX‐A and the placebo‐treated groups, all subjects except one in each group had bilateral myalgia. All subjects were diagnosed with masseter muscle myalgia and 95% and 90%, respectively were also diagnosed with myalgia of the temporalis muscle in the BTX‐A and placebo group.

Secondary TMD diagnoses were also registered where 46% in the BTX‐A group were diagnosed with arthralgia compared with 45% in the placebo group. Additionally, 25% in the BTX‐A group had disc displacement with reduction, compared with 36% in the placebo group. Possible bruxism was found in 58% of subjects in the BTX‐A group and 50% in the placebo group. Finally, 75% of subjects in the BTX‐A group had headache associated with TMD, compared with 59% in the placebo group.

### 3.1 Efficacy

The primary efficacy measure *change in number of days with pain at jaw function* decreased from a median (interquartile range) of 14 (4) to 10.5 (6) (*p* = 0.018) in the BTX‐A group and from 14 (1.5) to 14 (5) (*p* = 0.168) in the placebo group. There was no statistically significant difference between the groups (*p* = 0.585). Number of days with jaw pain at rest were significantly fewer in both groups but not significantly between the two groups (Table 2).

**TABLE 2:** Diary registrations and point estimated pain immediately before intervention and at evaluation visit. ITT population.

	Botox				Placebo				*p* between groups
	*n* ^§^	Baseline	Evaluation	*p*	*n* ^§^	Baseline	Evaluation	*p*	
Pain registered in a diary									
Number of days with jaw pain at function^a,‡^	24/24	14 (4)	10.5 (4)	0.018	22/22	14 (1.5)	14 (5)	0.168	0.585
Number of days with jaw pain at rest^a,^ ^‡^	24/24	14 (1.7)	13.5 (8.2)	0.010	22/22	14 (3.2)	12.5 (7.2)	0.068	0.861
Number of days with pain at maximum opening^b,^ ^†^	24/21	61.6 (29.8)	44.9 (29.7)	< 0.001	20/21	65.4 (28.4)	52.8 (34.1)	0.008	0.816
Point estimated pain at baseline and evaluation visit at the clinic									
Pain at maximum opening^b,^ ^†^	24/23	4.8 (2.3)	3.6 (2.3)	0.004	22/22	4.7 (2.4)	3.5 (2.3)	0.001	0.948
Pain at jaw rest^b,^ ^†^	24/23	3.9 (1.5)	2.8 (1.9)	0.027	22/22	3.4 (1.8)	2.0 (1.7)	< 0.001	0.696
JFLS‐8^a^	24/23	8.5 (12.5)	8 (9)	0.030	22/22	12 (17.7)	5.5 (12)	< 0.001	0.260
PHQ‐9^a^	24/21	5.5 (6.7)	3.0 (2.5)	0.002	22/21	5.0 (7.5)	4.0 (6)	0.137	0.487
GCPS characteristic pain intensity^b^	24/23	5.0 (1.7)	3.6 (1.8)	0.003	22/22	4.6 (2.2)	3.0 (1.9)	< 0.001	0.530

*Note:* Data are ^a^median (interquartile range) or ^b^mean (SD). Differences within groups tested by ^a^Wilcoxon Signed Rank Test or ^b^Paired *t*‐test and between groups by ^a^Mann–Whitney *U*‐test or ^b^Student's *t*‐test. The tests of differences within groups compare the values at baseline and at evaluation.

Abbreviations: GCPS, Graded Chronic Pain Severity Scale; JFLS‐8, Jaw Functional Limitation Scale; PHQ‐9, Subject Health Questionnaire‐9.

^§^ Number at baseline/number at follow‐up.

^‡^ Number of days with jaw pain during 2 weeks before treatment and 2 weeks before evaluation visit.

^†^ Numeric rating scale (0–10) with the end definitions ‘No pain’ and ‘Worst pain imaginable’.

**FIGURE 3:**
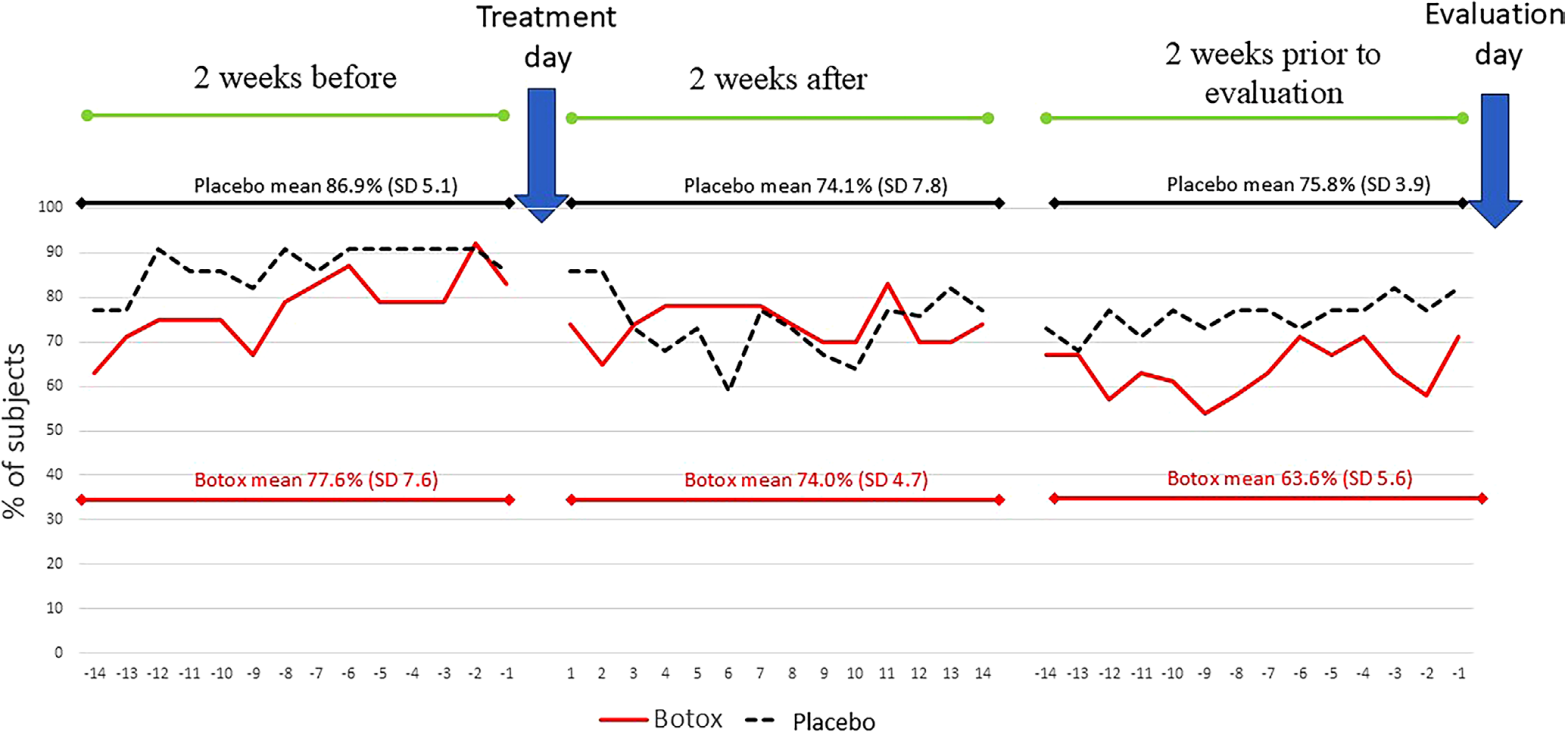
Proportion of subjects reporting pain at jaw function in a questionnaire during 2 weeks before and after the intervention and during the 2 weeks prior to the evaluation visit.

The proportion of the subjects' diaries reporting jaw functional pain during the 14 days before and after intervention as well as the 14 days before evaluation visit is graphically illustrated in Figure 3. At baseline the NRS_point estimate_ pain score at jaw rest and at maximum opening did not show any significant differences between the groups. At evaluation visit, these pain measures decreased but without significant difference between groups (Table 2). The proportion of study subjects who achieved ≥ 50% reduction in number of pain days during jaw function was low, that is, 29% and 18% for BTX‐A and placebo, respectively (*p* = 0.383). Absolute risk reduction was 11% and the number needed to treat (NNT) 9.1.

Jaw function evaluated with the JFLS‐8 instrument revealed significant improvement in the total score in both the BTX‐A and placebo‐treated group, but with nonsignificant difference between groups (Table 3). Baseline PHQ‐9 values were low and at follow‐up the BTX‐A group showed significant improvement although no significant difference between groups (Table 2).

**TABLE 3:** Mean change (in mm) of jaw opening capacity and proportion of subjects with uni‐ or bilateral palpation tenderness of temporalis/masseter muscles and the temporomandibular joint at baseline and at evaluation visit 2 months after intervention.

	Botox (*n* = 23)			Placebo (*n* = 22)			Botox vs. placebo
	Baseline visit	Evaluation visit	*p*	Baseline visit	Evaluation visit	*p*	*p*
Maximum opening without pain^a^	37.7 (12.5)	41.2 (10.5)	0.047^a^	37.0 (9.5)	42.9 (10.6)	0.001^a^	0.288^a^
Maximum opening with pain^a^	49.9 (8.2)	50.5 (9.0)	0.580^a^	47.8 (6.1)	50.8 (4.8)	0.010^a^	0.160^a^
Maximum opening with assistance^a^	53.1 (7.8)	52.9 (8.4)	0.818^a^	51.7 (5.9)	53.4 (5.0)	0.065^a^	0.173^a^
Palpation tenderness of TMJ^b^	14/24 (58)	12/23 (52)	0.754^b^	13/22 (59)	9/22 (41)	0.344^b^	0.449^b^
Palpation tenderness of m. temporalis^b^	22/24 (92)	18/23 (78)	0.375^b^	20/22 (91)	15/22 (68)	0.063^b^	0.445^b^
Palpation tenderness of m. masseter^b^	24/24 (100)	20/23 (87)	0.250^b^	21/22 (95)	17/22 (77)	0.125^b^	0.459^b^

^a^ Mean (SD). Differences within groups between baseline and evaluation tested by Paired *t*‐test. Differences between groups tested by Student's *t*‐test.

^b^ *n*/*N* (%). Differences within groups between baseline and evaluation tested by Related‐Samples McNemar change test. Differences between groups tested by Fisher's exact probability test (improved vs. worsened from baseline to evaluation were compared).

Responders defined as ‘much improved’ or ‘very much improved’ on the PGIC scale were 26% and 32% in the BTX‐A and the placebo group, respectively. The proportion of responders (pain relief ≥ 30%) among BTX‐A and placebo treated, respectively, were 48% and 64% (*p* = 0.286) for the measure ‘pain right now’, 57% and 59% (*p* = 0.862) for ‘pain as a mean past month’, and 35% and 46% (*p* = 0.465) for ‘worst pain the past month’.

The GCPS rating of characteristic pain intensity changed significantly within each treatment group but with nonsignificant difference between groups (Table 2). The maximum jaw opening capacity with and without pain as well as opening with assistance increased in both groups but with no significant difference between groups (Table 3). Palpation tenderness over the masseter and temporalis muscles or TMJ did not change over the course of treatment time (Table 3). The use of analgesics registered in the diary did not change in any of the two groups.

### 3.2 Adverse Experiences

The incidences of reported and observed treatment‐related adverse experiences were few (five in the BTX‐A group and six in the placebo group), and quickly resolved, with no preponderance to any group. The subject excluded after randomisation did not report any adverse events. Adverse events included fatigue, headache, nausea following injections, feeling of being about to faint, fever, vertigo and thrill in the jaw.

## 4 Discussion

The IMMPACT recommendations were established for interpreting the clinical important treatment outcomes in pain trials [19] and recommend four outcome domains: pain intensity, physical functioning, emotional functioning and participants' ratings of overall improvement. In our study, all four domains were evaluated using the CPI, JFLS‐8, PHQ‐9 and PGIC scales and all showed nonsignificant differences between BTX and placebo treatments.

The primary efficacy measure in this study, the number of days with jaw pain during function, showed a greater median numeric reduction in the BTX‐A group compared with the placebo group. The results indicate that BTX‐A tends to reduce the median number of days with pain by approximately 25%, although nonsignificant to the effect of placebo. These findings are consistent with previous studies [20, 21].

Based on a post hoc power analysis (80% power, significance level 0.05) based on data from the present study, a number of 67 + 67 subjects plus replacements is required to show a significant benefit of BTX‐A. The umbrella diagnosis of myalgia was mostly used for diagnosing subjects in the present study, including those with myofascial pain with referral. These subjects may have different pathogeneses and, consequently, varying sensitivity to treatment. Therefore, it could be of value to have a more homogeneous group in future studies. At enrolment in our study, the subjects reported a mean pain intensity of 3.9 and 3.4 on the 0–10 NRS scale in the BTX‐A and placebo group, respectively. This indicates a relatively low level of pain, especially considering that these patients were selected from specialist clinics. Our study subjects reported much lower pain scores than previously reported in a recent BTX‐A study where median pain intensities of 70–74 (100 graded VAS scale) were reported and significant effects found [22]. Future studies should consider the fact that it is easier to show potential benefits with higher baseline pain scores.

Among several indications, BTX is indicated for the treatment of chronic migraine. One of the key efficacy variables for the migraine indication is ‘change from baseline in frequency of headache days’. In the US packet insert [16], a mean significant difference in 1.4 and 2.3 headache days was registered between BTX‐A and placebo in two pivotal studies. In our jaw myofascial pain study, the median change of pain days was greater, but not significant. The reason is likely that our study did not have sufficient power to demonstrate significant difference. The NNT was also unsatisfactory, at 9.1 in the present study, but slightly better compared with the Ernberg et al. study [21] where the NNT at a 1‐month follow‐up was 11.

A Cochrane review of BTX‐A effects on myofascial pain outside the head and neck area showed inconclusive evidence to support the use of BTX‐A in that treatment [23]. Langevin et al. [24] concluded in another Cochrane review that evidence fails to confirm either a clinically important or statistically significant benefit of BTX‐A injection for chronic neck pain associated with or without cervicogenic headache. A randomised controlled trial of myofascial TMD pain [21] also failed to show any pain‐relieving benefits, results that are confirmed in the present study.

BTX‐A treatment of masseter hypertrophy was evaluated by Fedorowicz, van Zuuren, and Schoones [25] in a 2013 Cochrane report. They were unable to identify any RCT study treating masseter hypertrophy and in the absence of high‐level evidence they recommended well‐designed, adequately powered RCTs. In a review from 2022, Delcanho et al. [26] concluded that ‘the data to date justify further research to better identify the patients most likely to benefit and to establish the best protocol (e.g., site, number, and dose of injections) for treating different TMDs (e.g., myofascial pain, TMJ osteoarthritis, and internal derangement). Such research is required before routine clinical use of BTX‐A to treat TMDs can be recommended’. But in the authors general conclusion it was stated that ‘there is good scientific evidence to support the use of BTX‐A injections for treatment of masseter hypertrophy’. Recent studies also show a reduction of muscle thickness after injections with BTX‐A [27].

All the secondary measures in the present study showed small clinically nonrelevant effects and nonsignificant differences between the active substance and the control. The use of analgesics remained high in both groups and at the same level before treatment as well as directly after treatment and at follow‐up. The consistent high usage of analgesics in both groups may suggest a potential confounder on subjects' pain experience. The present study was exploratory and of all variables tested, the number of days with jaw pain at function seems to be useful to test in upcoming fully powered studies.

The BTX‐A incidence of reported and observed treatment‐related adverse experiences was low and of the same magnitude as the placebo treatment. No serious adverse events were seen and to conclude from this small study, BTX‐A treatment is safe. However, consideration must be taken regarding the innervation of the face and jaw structures, and the treatment requires comprehensive knowledge of the anatomical structures. The systematic umbrella review by De la Torre Canales et al. [13] highlights a higher risk for adverse events on muscle and bone tissue from BTX‐A treatment.

In Sweden, BTX‐A is approved for the treatment of ankle, foot and wrist spasticity in cerebral palsy after stroke, in blepharospasm, hemifacial spasm and associated focal dystonia, cervical dystonia, and as symptom relief in chronic migraine. In addition, the drug is used off‐label for a series of conditions, including myofascial pain, and there is an indication that BTX‐A has a significant therapeutic effect in reducing bruxism episodes [28].

The placebo or procedural effects in injecting substances into the masticatory structures may be profound. In a randomised controlled trial with intra‐articular injections of corticosteroid in the jaw joint for the treatment of arthralgia, the placebo had the same positive pain‐relieving results as the active substance [29]. However, it is difficult to conclude any placebo‐ or procedure‐related benefits in our study, as the level of complaints remained at the same level as before treatment.

The major strength of this study is the study design, that is, randomised, controlled and double‐blind with the use of all the IMMPACT recommended measures. Another strength was the replacement of the traditional primary measure ‘pain intensity’ with the use of ‘change in number of days with pain at jaw function’, which showed a numerically greater effect in the BTX‐A group compared with the placebo group. The major limitation of the study is the lack of power to show a significant BTX‐A effect. Other limitations were the very long time for inclusion of study subjects, lack of knowledge of lowest effective dose, and the number of optimal injection sites. Another drawback was the extensive use of analgesics throughout the trial period. Patients with myofascial pain may also have different pathogenesis resulting in several known and unknown confounders that may interact with the study outcome.

The National Board of Health and Welfare in Sweden does not recommend the use of BTX‐A for treating myofascial jaw pain because of insufficient scientific evidence [30]. The result of the present study supports that recommendation. However, whereas previous studies on BTX‐A efficacy have predominantly used pain intensity reduction as the primary measure, an approach using ‘reduction of days with functional jaw pain’ might be a more fruitful way to illustrate the benefits of BTX‐A treatment.

## 5 Conclusion

While a single treatment of BTX‐A injections in the masseter and temporalis muscles was not effective in treating jaw muscle myalgia, an adequately powered study might provide sufficient support for efficacy.

## Author Contributions

A.M. developed the research question, contributed to the study protocol development, arranged funding, participated in manuscript preparation, enrolled and treated subjects. H.A., P.A., E.L. and D.O. arranged funding, reviewed in manuscript content, enrolled and treated subjects. N.Y. prepared manuscript, enrolled and treated subjects. E.N. was the data manger, responsible for the statistical analysis and data interpretation and reviewed the manuscript. G.I. contributed to the study design, protocol development, arranged funding, was the contact person to authorities and prepared the manuscript.

## Conflicts of Interest

All authors certify that they have no affiliations with or involvement in any organisation or entity with any financial interest (such as honoraria, educational grants; participation in speakers' bureaus; membership, employment, consultancies, stock ownership or other equity interest and expert testimony or patent‐licensing arrangements), or nonfinancial interest (such as personal or professional relationships, affiliations, knowledge or beliefs) in the subject matter or materials discussed in this manuscript.

The study funders had no role in the study design, collection, interpretation or analysis of the data; report writing, or the decision to submit the paper for publication.

### Peer Review

The peer review history for this article is available at https://www.webofscience.com/api/gateway/wos/peer‐review/10.1111/joor.13915.

## Data Availability

The data is available to the public under an open access license. Additional information regarding data availability can be obtained by contacting the corresponding author. The data that support the findings of this study are openly available in [repository name e.g “figshare”].
